# Exploring the challenges and opportunities towards optimal breastfeeding in Ethiopia: a formative qualitative study

**DOI:** 10.1186/s13006-020-00265-0

**Published:** 2020-03-30

**Authors:** Kidane Tadesse Gebremariam, Oksana Zelenko, Znabu Hadush, Afework Mulugeta, Danielle Gallegos

**Affiliations:** 1 School of Exercise and Nutrition Sciences, Queensland University of Technology, Brisbane City, QLD Australia; 2 School of Public Health, College of Health Sciences, Mekelle University, Mekelle, Ethiopia; 3 School of Design, Queensland University of Technology, Brisbane City, QLD Australia; 4 Centre for Child Health Research, Institute of Health and Biomedical Innovation, Brisbane City, QLD Australia

**Keywords:** Exclusive breastfeeding, Focus groups, Parents, Perceptions, Attitudes, Barriers, Ethiopia

## Abstract

**Background:**

Breastfeeding, particularly exclusive breastfeeding, is essential to ensure the short- and long-term health of infants and mothers. Sub-optimal breastfeeding practices currently take place in low income countries contributing to morbidity and mortality. This research explored the challenges and opportunities around exclusive breastfeeding in a large city in Ethiopia to inform a larger breastfeeding intervention.

**Methods:**

Fathers and mothers who had children less than 2 years of age, and who could speak, and understand Tigrigna were recruited from two health centres located in Mekelle, Ethiopia. Two focus group discussions (FGDs) with fathers and two FGDs with mothers (*n* = 42) were conducted using a semi-structured interview guide to explore the challenges and opportunities related to breastfeeding. Discussions were audio-recorded and transcribed in Tigrigna and translated to English. The data were manually analysed using thematic analysis, generating open codes which were grouped to form themes.

**Results:**

Four themes with 11 sub-themes emerged. The themes identifies were: conflicted emotions on the birth of baby (feeling happy and feeling worried); perspectives on intergenerational approaches (old-fashioned beliefs of grandparents and the power of science, breastfeeding in public, breastfeeding knowledge); gender roles as barriers and enablers (the burden on women, changes in men’s roles and the financial status of the household); the role of healthcare (delivery of health information to parents, the role of health care providers and perceptions of inadequate milk supply).

**Conclusion:**

Parents reported a range of opportunities with respect to breastfeeding, including the power of a scientific approach, the positive role of healthcare, and shifts within gender roles that maximized the potential support from fathers. However, there remains a tension between the beliefs of older generations and current best-practice. Parents continue to need ongoing support in order to practice optimal breastfeeding.

## Background

Best practice breastfeeding, includes early initiation (within 1 hour of birth), exclusivity (only breastmilk) until 6 months of age, and continuation with appropriate introduction of complementary foods until 2 years of age [1]. These optimal infant feeding practices, contribute to the prevention of 823,000 annual infant deaths worldwide, with most occurring in low income countries [2]. Globally, sub-optimal infant feeding practices (late initiation, short duration and non-exclusivity) contributes to high rates of infant mortality and morbidity [3, 4]. In Ethiopia, breastfeeding an infant for at least some time whether exclusively or not, is nearly universal [5]. However, early initiation of breastfeeding, within 1 hour of birth, occurs only in about one-half of infants (51.5%) and just over one-half (57.5%) of infants are exclusively breastfed (EBF) at 6 months of [5].

The low level of EBF in Ethiopia is primarily due to the administration of fluids such as plain water, non-human milk, sugar dissolved in water, and juice in addition to breastmilk (known as pre-lacteal feeds), and other foods introduced to infants at an early age [6, 7]. Taking into consideration that 27–43% of infants in Ethiopia are exposed to this range of foods and liquids [6, 8, 9] in the first few months of life, the current rate of EBF at 6 months could be an over-estimation. Acknowledging this low level of EBF, the Ethiopian government had planned to improve the level of EBF at 6 months to 70% by 2015 [6]. However, in the absence of national data, findings from smaller studies from throughout Ethiopia indicate that current EBF levels remain considerably lower than the identified national prevalence and targets [10–12].

Psychosocial factors, including maternal intention to breastfeed, breastfeeding self-efficacy, knowledge, attitudes and social support, are associated with the early initiation and continued EBF [13, 14]. Similarly, in low income countries mothers with better self-efficacy, intention to breastfeed, positive attitudes towards breastfeeding, good breastfeeding knowledge, and who have access to support are more likely to initiate breastfeeding early and exclusively breastfeed for 6 months [15–17]. Fathers’ beliefs on whether their partners should breastfeed, strongly predicts maternal intention to breastfeed. Additionally, mothers who perceive that their partners prefer breastfeeding are less likely to cease breastfeeding at any time compared to those who perceive their partners prefer bottle-feeding, or are ambivalent about how their child is fed [18].

In Ethiopia, there have been indications that fathers are aware of the importance of EBF and are ready to support their partners to breastfeed [19]. Fathers have identified areas where they could assist their partners including providing advice on nutrition during the breastfeeding period; providing food and financial support to the family; providing information about healthy eating for the mother and the infant; and sharing the housework. The factors identified that contributed to fathers being supportive around breastfeeding included: their role as head of household; love for their partner; and the desire for a healthy and intelligent child. The most commonly identified barriers were a lack of specific knowledge about infant and young child feeding, and gender issues related to women’s roles outside the home where mothers need to work in paid employment to contribute to the household income, thus limiting their ability to exclusively breastfeed [20].

A number of different breastfeeding education interventions have been shown to be effective in improving early initiation, exclusivity to 6 months and continuation of breastfeeding [21–27]. Breastfeeding is impacted by a range of contextual factors which vary depending on socio-economic [11, 28], and psychosocial factors [13, 14], as well as the presence or absence of social support [1, 18, 29]. Therefore, it is imperative that the challenges and opportunities of breastfeeding within communities is articulated in order to inform breastfeeding interventions for maximum effect. Thus, this qualitative study was designed to explore the challenges and opportunities related to breastfeeding to inform the development of a Short Message Services (SMS) text messaging based breastfeeding education intervention, to improve EBF in Mekelle, Ethiopia.

## Methods

### Setting and design

This research took place in two health centres located in Mekelle, Ethiopia. Mekelle is a large city with a population of 412,938 located in north Ethiopia. Focus group discussions (FGD) were used to investigate individual and community-level perspectives around breastfeeding [30]. This formative qualitative study was conducted in February 2018 to explore challenges and opportunities towards EBF. Four separate FGDs were undertaken, Two with mothers and two with fathers.

### Participants

Potential study participants met the following inclusion criteria: (i) had at least one child less than 2 years of age; (ii) lived with their partner; (iii) were able to speak and read Tigrigna. Potentially eligible participants were approached at each health centre or during house-to-house visits by an urban health extension worker. For eligible participants, health extension workers verbally provided detailed information about the study prior to obtaining written informed consent. The aim was to recruit approximately 12 participants to each focus group [31]. Health extension workers negotiated with consenting participants regarding the date and time for the FGDs. All discussions took place at the respective health centres, with FGDs for fathers occurring in the evening. All participants received BIR300 (approximately 10USD) to compensate them for their time and any travel expenses.

### Data collection

A semi-structured FGD interview guide was developed after a review of the literature [19, 32–36]. The questions were under the following categories: breastfeeding knowledge, attitudes, self-efficacy, and partner support. The discussions were conducted in a private room, in order to maintain participant privacy and to enable audio-recording with minimal disruption. The principal investigator assisted by an experienced, trained male data collector conducted the FGDs for fathers while two experienced, trained female data collectors conducted the mothers’ FGDs. FGDs followed established guidelines that meant ground rules were first discussed to facilitate the establishment of trust, minimize overtalking and reiterated the importance of all voices to be heard. Facilitators reminded participants that there were no right or wrong answers and created an environment for participants that mimicked a casual conversation with their peers. Participants introduced themselves and discussion commenced. Probes were used to elicit further information and to motivate participants to share their views. Discussions ran for up to 2 hours and were audio-recorded. Field notes provided additional information and clarification if the audio-recording or points discussed were unclear.

### Data analysis

The audio-recorded data were first transcribed in the local language (Tigrigna) immediately after completion of each FGD, and then translated into English by the principal investigator (KTG). The English transcripts were analyzed independently by KTG and ZH, and DG and OZ using line-by-line reading in order to generate initial open codes or labeling concepts. Any transcription that was not clear in English was discussed between KTG and AM. KTG and DG categorized open codes into themes; similar themes were merged, and divergent themes discussed between all authors until consensus was reached. The text relating to the identified themes was described and summarized using verbatim quotes [37].

## Results

A total of four FGDs were conducted involving 21 mothers and 21 fathers. Each focus group had between 10 and 11 participants. The demographic characteristics of the participants are presented in Table 1.

**Table 1:** Characteristics of focus group participants (*n* = 42)

Variable	Number (%)
**Sex**	
Male	21 (50)
**Educational status**	
No formal schooling	4 (9.5)
Primary	20 (47.6)
Secondary	11 (26.2)
University	4 (9.5)
Not specified	3 (7.2)
**Number of children**	
1–2	22 (52.4)
3–4	14 (33.3)
5 or more	6 (14.3)
**Age**	Mean age (yrs) + SD
Male	37.8 + 9
Female	30 + 5.2

Four key themes related to breastfeeding were identified: (1) conflicted emotions on the birth of a baby; (2) the importance of a scientific approach; (3) gender roles as barriers and enablers; (4) the role of healthcare; and (5) barriers to breastfeeding (Table 2). No new themes were emerging by the final FGD and so saturation was achieved.

**Table 2:** Summary of identified themes and sub-themes from focus group discussions

Themes	Sub-themes
Conflicted emotions on the birth of baby	- Feeling happy - Feeling worried
Perspectives on intergenerational approaches	- Old-fashioned beliefs of grandparents and the power of science - Breastfeeding in public - Breastfeeding knowledge
Gender roles as barriers and enablers	- Burden on women - Change in men’s roles - Financial status of the household
Role of healthcare	- Delivery of health information - Health care providers - Perceptions of inadequate milk supply

### Theme-1: conflicted emotions on the birth of baby

#### Feeling happy

Participants’ emotions on the birth of a child ranged from joy to worry. Participants described the birth of a child as “a gift from God”. There was a strong belief that having children contributes to a meaningful life. In contrast, if you did not have children you could be regarded as selfish.

“I am very happy! Because a child is something that makes your life meaningful. It is quite wonderful, a change from one to two, it gives you happiness because you bring someone you can talk to, I am very happy.” *(M1.5)*

“When the baby is born, you feel like you reborn. Because it is not good to live alone, babies are good. When you live alone, society will say you live for yourself, which means a meaningless life. The baby is part of you and your happiness, a baby means big happiness. Except for the economic issues, the society wants us to have many children.” *(F1.6)*

#### Feeling worried

However, while children were considered a joy, pregnancy and childbirth were also times of great stress and worry. Fathers worried about the health of their partners during pregnancy and childbirth. Due to past experiences fathers felt strongly that delivery should take place at health facilities to minimize any problems that may arise during childbirth. However, none of the mothers expressed similar concerns associated with pregnancy and childbirth.

“Because you see many problems happening with other mothers, there are many problems, especially when she is a first-time mother, many things happen. But today there is a lot of technology, thus, many problems are not happening. When your wife has a healthy birth, you feel happy. But when you see problems with your wife or other mothers you feel worried about what is going to happen*.” (F1.4)*

“You worry very much starting from pregnancy until delivery. You hope she will give birth safely, you feel worried even when she is at a health facility until she gives birth. But when the baby is born, and you hear the baby crying you feel happy. You feel happy when the baby and the mother come home healthy.” *(F2.4)*

### Theme-2: perspectives of intergenerational approaches

#### Old-fashioned beliefs of grandparents and the power of science

Participants acknowledged the tension between what they considered modern breastfeeding knowledge and old-fashioned beliefs expressed by grandparents. Modern breastfeeding was equated to the scientific approach as espoused by health professionals. However, participants were reluctant to challenge grandparents about their beliefs in case it led to tension.

“In society it was believed that a baby should start additional foods when the baby starts smelling [smells the food]. This was the belief of our parents. Now I get information from the health facility, and from different books that state babies should be fed only the mother’s breastmilk for the first 6 months.” *(F2.6)*

“For instance, my baby cries always, and my mother says give him water, I say please do not give him any water! She says ok you want to kill him, kill him! Then if something happens, you feel like he is not getting water from the breastmilk, then I feel worried.” *(M1.4)*

“Because our parents did not know the current science and did not follow that, they do not accept science. They may have some influence, but we follow what we believe.” *(F2.3)*

Participants indicated that they thought the old beliefs were harmful. Some of these beliefs included; giving water to an infant when they were crying, giving fenugreek for infant abdominal pain, avoiding the evil eye by not breastfeeding in public, and the belief that if the mother breastfed while pregnant the infant would develop an enlarged abdomen. The participants identified that the beliefs expressed by their parents about how they take care of them during their childhood, and grandparents felt that these practices should be continued with their grandchildren.

*“*At home I have encountered when the baby gets sick due to abdominal pain, grandparents say give him fenugreek. Most of the people saying this are older than me.” *(F2.3)*

“Old family members told us to provide foods to the infant before six months, they tell you their experiences of giving food earlier. My mother was telling me to give food before six months, but now we start additional foods after six months.” *(M2.4)*

#### Breastfeeding in public

Breastfeeding in public was a divisive issue. Fathers held both ambivalent and negative views regarding breastfeeding in public. A number of fathers indicated that women should not breastfeed in public due to the evil eye creating sickness in the infant and that other people watching would reduce the production of breastmilk.

“I will not be happy, not only in public but even at home. If there are other people, it is not good because the baby will be affected evil eye ”*(F2.8)*

“I will not be happy! When the baby is breastfed in front of another person, I do not feel the baby is getting enough.” *(F1.7)*

On the other hand, most mothers and some fathers did not see any issues about breastfeeding in public. They expressed the belief that infants should be fed when they are hungry regardless of location. The issue around infants becoming unwell if breastfed in public was also associated with poor air quality in public places.

“I have a child, when I go to the market or other places if she cries or wakes up it is a must to breastfeed her whether there are people or not. I cannot keep quiet while she is crying, whether there are people or not, breastfeeding must not be stopped. If not, this will affect the baby.” *(M1.6)*

“I will not feel bad if she breastfed the baby at any place. But when you see practically, when mothers bring the babies with them to a social gathering, it could be due to the sun, but the baby feels like gastritis and feels discomfort.” *(F2.2)*

“The public is not a problem, but we do not want the baby to be exposed to the heat of the sun while breastfeeding, so that, we go to seek shade from the sun and breastfeed. Apart from this breastfeeding in public is not a problem.” *(M2.8)*

#### Breastfeeding knowledge

Parents indicated that the information they received from health professionals, health facilities and provided on the radio had moved away from older infant feeding practices, particularly those that encouraged discarding colostrum and introducing additional foods and liquids at an early age. However, the parents taking part in the focus groups were able to identify the three recommended infant feeding practices: putting the infant to the breast within 1 hour of birth; EBF for the first 6 months; and continued breastfeeding with the addition of foods until the age of 2 to 3 years.

“Feeding a baby only breastmilk for the first 6 months is becoming our culture due to the information we got from the health facility and the house-to-house visits from the health extension workers. Our current practice is only breastmilk, no other additional foods for the first 6 months.” *(F1.6)*

“There are home visits from the health center, in addition when the baby is not well, we go to the health center and they told us not to feed anything other than breastmilk before 6 months. Some people say when the baby is fed with breastmilk, he/she will get thirsty so that the baby should drink water; for the first 6 months I have never done this myself.” *(M1.3)*

Participants described exclusively breastfed babies as having higher intelligence, physically stronger and protected from illness. Exclusively breastfed babies were described as much healthier than babies who started additional foods or liquids before 6 months of age.

“Exclusively breastfed babies become intelligent. If we take care and breastfeed them, we can protect them from illness.” *(M1.6)*

While the benefits for the infant were well-known, participants expressed a different view regarding the benefit of breastfeeding for mothers. There were strong indications that breastfeeding was detrimental to a mother’s health and appearance. Both mothers and fathers said that, although breastfeeding was good for the baby it did not benefit the mother. Mothers who cease breastfeeding before 6 months were described as having a better appearance compared to mothers who continued breastfeeding.

“The baby who is fed with breastmilk for 6 months will have better health. If she breastfeeds for 6 months, it will be good for the health of the baby but not for the mother.” *(F1.1)*

Fathers also indicated that while they believed that breastfeeding was detrimental to mothers, they had a strong appreciation for mothers who exclusively breastfed their babies for the full 6 months.

### Theme-3: gender roles as barriers and enablers

Participants acknowledged that each gender had a role to play in supporting breastfeeding. Mothers and fathers indicated that women were mainly responsible for housework including food preparation and taking care of children. Support was provided by grandmothers and, in some cases, housemaids. Mothers who were government employees were more likely to have a housemaid who assisted with childcare and housework. However as described later, returning to work also impacted negatively on EBF. The role of the father was as a provider, the expectation being that they were responsible for financially supporting the family and enabling the purchase of food that would contribute to the health of the mother and their children.

Some fathers described participating in meal preparation and serving the family particularly when mothers were breastfeeding. Mothers indicated the expectation that fathers should participate in caring for children and undertaking housework in addition to providing financial support.

“The husband and wife are one. If the baby cries either you take care of the baby, or you cook and allow her to breastfeed the baby and serve a meal to your wife. For example, if the baby cries, I cook myself so to help my wife, it is not like in previous times.” *(F1.8)*

“If you have the capacity, you can have your own housemaid and get support, if you do not have the capacity and if your husband has to go to work in the morning, he should help you by doing some of your activities before he goes to work.” *(M2.4)*

“It is the [fathers] responsibility to provide food and support. [Fathers] should take care of the baby, because it is part of you and you cannot keep quiet while the baby is crying because it makes you worried. Previously, fathers were not expected to take care of children it was the responsibility of the mothers, but now due to science and education we are all aware and take care of our children.” *(F1.9)*

The working status of the mother impacted on the household financial situation, which in turn impacted on access to food and beverages for the breastfeeding mother and how much work (paid and unpaid (housework) the mother had to undertake. Wealthier households were able to afford housemaids to assist with childcare and housework and/or were able to stay home from paid work for longer periods of time. Women who had to return to work often left their infant in the care of housemaids, grandmothers or neighbors. Expressing breastmilk is not a common practice and consequently the infant was often fed liquids and foods other than breastmilk. One of the significant barriers identified by participants was the lack of paid maternity leave. Mothers working in government offices only receive 3 months maternity leave, making EBF for 6 months difficult.

“Mothers working in the government offices, go to work at 8am and return home at 5pm, which means in between this time she will not be able to breastfeed her baby, because she must go to work. She does not have an option and so she leaves the baby either powder or cows’ milk.” *(M1.4)*

“When the mothers want to go out to do something or go to work, they leave the baby with their neighbors. When the baby cries then she [the neighbor] will give [the baby] water or other foods.” *(F1.6)*

“Mothers want to breastfeed, but if they do not have enough food and have economic problems, they might not breastfeed the way they want. The second thing is, when the mother is busy with activities at home she will not remember. There is an interest to breastfeed; the problem is we start to do something and then we forget to breastfeed the baby.” *(M2.7)*

### Theme-4: role of healthcare

#### Delivery of health information and health care providers

Most participants attributed positive changes in attitudes toward breastfeeding practices to the efforts of health care providers. They also indicated strong trust in the scientific approach adopted by health professionals and the personalize services and information they provided. The comprehensive antenatal and postnatal follow-up provided by health facilities contributed to the improvement in knowledge regarding best practice infant feeding.

“Health professionals tell us we should not have to give cow’s milk and other foods in the first 6 months.” *(M2.11)*

“We got education from the health extension workers. In addition, before delivery, we got health education at the antenatal clinic follow-up. After delivery they check on what we feed out babies [and] provide the monthly immunization. We get good follow-up. Because of the good follow-up and the information, we are able to take care of our children” *(M1.2)*

Fathers also described the more recent role of healthcare services in minimizing pregnancy complications and the risk of poor childbirth outcomes for both mothers and infants.

“It is better to deliver at a health facility than a home delivery, to get your baby healthy and your happiness.” *(F2.4)*

#### Perceptions of low milk supply

Despite the support from healthcare providers participants still identified perceived inadequate milk supply as a barrier to exclusive breastfeeding. Inadequate milk supply was one of the most commonly cited factors explaining the early introduction of additional liquids or foods before 6 months. Mothers and fathers described lack of adequate milk supply as an outcome of not being able to produce enough milk.

“It could be due to lack of enough breastmilk; in this case the mother gives the baby additional food or water because the baby cries.” *(F1.6)*

“When there is not enough milk, if she could not produce milk could make breastfeeding difficult.” *(M2.1)*

## Discussion

The current study aimed to explore the challenges and opportunities identified by parents regarding EBF in Mekelle, Ethiopia in order to inform the development of messages for a SMS-based intervention. These parents identified a shifting belief system with respect to breastfeeding that was informed by information provided by health services. This information created tensions with the older generation who still believed in certain cultural feeding practices, which were inconsistent with international guidelines. Parents also identified a range of individual and structural barriers that impacted on breastfeeding behaviors.

The current study highlights a number of opportunities with respect to breastfeeding practices in Ethiopia. The first is the more active role played by Ethiopian fathers in pregnancy and childbirth. The encouragement from fathers to adopt a “more scientific” approach including giving birth in a health service, helped to ameliorate some of the concern associated with the birthing process. Study from Ethiopia that revealed mothers whose partners were actively involved in decision-making on maternal and newborn child health, were found to deliver in health facilities at the hands of skilled birth attendants [38]. In addition, these fathers continue to develop an active role by participating in birth preparedness, choosing a health facility for delivery, accompanying their wives during labor, and financing health-related bills [39]. This ongoing active role is indicates that fathers could potentially influence breastfeeding practice and should be included in any intervention facilitating optimal breastfeeding [36].

The efforts to improve breastfeeding knowledge via healthcare providers and public health messages appears to have been successful in raising awareness. Breastfeeding knowledge is known to have a significant effect on maternal breastfeeding attitudes and breastfeeding intentions [28]. According to a systematic review, parents in low-middle income countries (LMIC) who received breastfeeding education had improved breastfeeding knowledge resulting in improved breastfeeding initiation exclusivity and continuation rates [36]. However, raising awareness alone does not always lead to changed practices [26]; for example high awareness of EBF but sub-optimal implementation has been described in south-east Asia [40].

In this study the more modern breastfeeding practices espoused by healthcare providers were described as being a more “scientific approach”. This directly contrasted with the perceived beliefs of the older generation, which included practices that disrupted optimal infant feeding. Grandparents are known to influence breastfeeding practices especially in low income countries [41]. In this study the tensions that arose from the disparate viewpoints had the potential to contribute to sub-optimal practices, despite knowledge being high. Mothers and fathers need confidence to be able to counter cultural practices that are encouraged by the older generation. Interventions that target both parents may increase this dual self-efficacy and enable optimal infant feeding [26].

In line with previous studies [42, 43], this study showed that there are gender-specific roles related to breastfeeding practices. Women remain responsible for caring for the infant and other children, preparing meals and taking care of the household. While fathers are responsible for ensuring the financial capacity of the family in order to be able to purchase food and other important resources. However, there are indications that these gender-specific roles are more fluid with participants indicating more active involvement of fathers in food preparation, childcare and housework. Previous studies have indicated the importance of partners in supporting breastfeeding practice through motivation as well as practical assistance with childcare and housework [44]. Thus, mothers who have their partners’ support are more likely to exclusively breastfeed compared to non-supported mothers [45]. Therefore, breastfeeding interventions should include fathers and include practical suggestions on how they can support their partners as they breastfeed.

When exploring the challenges to breastfeeding, this study highlights universal barriers related to perceived insufficient milk supply and breastfeeding in public. These factors are known to interfere with maternal breastfeeding self-efficacy [46]. Previous studies have indicated that perceptions about adequate milk supply are strongly associated with EBF practices in both high and low-income countries [14, 47]. Perceived insufficient milk supply is among the most common reason mothers report for early breastfeeding cessation during the first few weeks postpartum [48]. Similarly, the current study indicated that insufficient milk supply is one reason for the introduction of prelacteal feeds. Underpinning the perception of insufficient milk is the fear that infants are not receiving adequate nourishment [48]. Studies have revealed that any form of infant crying or fussiness [49, 50] is considered a primary indication for perceived insufficient milk. Mothers with lower self-efficacy tend to doubt their ability to produce sufficient milk [50, 51].

Maternal breastfeeding self-efficacy is complex and plays a role in how mothers react emotionally to any difficulties related to breastfeeding [46]. Mothers who perceive they can successfully breastfeed were found to be problem-solvers and have positive thinking with greater determination to succeed. These mothers tend to consider difficulties as common compared to mothers who focus on the difficult aspects of breastfeeding [52]. Similarly, a study from Ethiopia showed that mothers with better breastfeeding self-efficacy were more likely to exclusively breastfeed at the fifth month [16]. Similarly, the current study indicated mothers’ self-efficacy is challenged by the old beliefs, and perception of insufficient milk supply. Thus, improving breastfeeding self-efficacy should be a component of future breastfeeding interventions.

Attitudes towards breastfeeding in public vary across geographic locations, cultures and religions [53]. Although mothers believe that breastfeeding is natural and unavoidable, in some instances they struggle to balance the needs of the infant and their embarrassment related to breastfeeding in public [54]. In this study, mothers expressed no embarrassment regarding breastfeeding in public. However, there were some indications that fathers did not support breastfeeding in public and this was influenced by a number of cultural beliefs. Fathers’ beliefs on whether their partners should breastfeed strongly predicts the level of maternal intention to breastfeed [18]. Moreover, maternal EBF behaviour is more strongly predicted by their partners’ breastfeeding beliefs than their own reasons to breastfeed [55]. Therefore, it is important that interventions include in their design messages that challenge fathers’ negative beliefs regarding breastfeeding in public, in order to support EBF.

While self-efficacy is an integral component contributing to optimal breastfeeding practice, environmental and policy factors are equally important. The current study shows that one of the factors hindering mothers from EBF was the time mothers were able to spend at home with infants in the first 6 months. Mothers from poorer households and who were government employees, were forced to go back to work before their infants are 6 months old. In contrast, those women who ran their own businesses were more likely to be able to breastfeed exclusively for 6 months. Previous studies have also revealed that the mother’s employment status impacts infant feeding practices because it determines how long the mother can spend with the infant during this critical period [14, 28]. Similarly, in Ethiopia, maternal employment status [56, 57], resumption to work before 3 months [12], and being a housewife [58, 59] determined the duration of EBF. Therefore, it is important to improve working conditions for women, creating enabling environments that facilitate EBF regardless of socioeconomic status. However, in the absence of policy change, behaviors that support EBF when the mother is separated from the infant should be promoted. In this case, messages should be developed that relate to leaving breastmilk at home and reminding housemaids, grandparents and neighbors that water and other foods/fluids should not be given prior to 6 months of age.

### Strengths and limitations

This formative qualitative assessment has strengths as well as limitations. First, as a qualitative study, it explored in-depth the existing challenges and opportunities of breastfeeding. Second, this study is one of only a few that have included the views of fathers to inform the design of future interventions. There are, however, a number of limitations. The purposive selection of participants from an urban area and included those willing to participate. The views therefore cannot be generalized across contexts. In addition, this study did not include grandparents who may be highly influential with respect to breastfeeding practice.

## Conclusion

This research has identified a range of opportunities with respect to encouraging breastfeeding, including following a more scientific approach that aligns with optimal feeding, the role of healthcare, and a shift in gender roles. However, there remains a tension between the beliefs of older generations and current best practice. In addition, structural barriers to breastfeeding were evident including lack of maternity leave and mothers needing to return to work to maintain an income. Parents in Ethiopia need continual support in order to practice ongoing optimal breastfeeding that integrates with the current health system but can be used within the home and accessible at any time. Therefore, interventions need to be developed that are easily accessible and provide messages to all family members.

## Data Availability

The datasets used and/or analysed during the current study are available from the corresponding author on reasonable request.

## Abbreviations

EBF: Exclusive Breastfeeding
FGD: Focus Group Discussion
LMIC: Low- and Middle-Income Country

## References

[1] World Health Assembly (2001). Global strategy for infant and young child feeding: the optimal duration of exclusive breastfeeding.

[2] Victora CG, Bahl R, Barros AJD, França GVA, Horton S, Krasevec J (2016). Breastfeeding in the 21st century: epidemiology, mechanisms, and lifelong effect. Lancet.

[3] Sankar MJ, Sinha B, Chowdhury R, Bhandari N, Taneja S, Martines J (2015). Optimal breastfeeding practices and infant and child mortality: a systematic review and meta-analysis. Acta Paediatr.

[4] Smith ER, Hurt L, Chowdhury R, Sinha B, Fawzi W, Edmond KM (2017). Delayed breastfeeding initiation and infant survival: a systematic review and meta-analysis. PLoS One.

[5] Central Statistical Agency (CSA) Ethiopia, ICF (2016). Ethiopia demographic and health survey 2016: key indicators report.

[6] CSA (2011). Ethiopia demographic and health survey report.

[7] Mulugeta A, Hagos F, Kruseman G, Linderhof V, Stoecker B, Abraha Z (2010). Child malnutrition in Tigray, northern Ethiopia. East Afr Med J.

[8] Bililign N, Kumsa H, Mulugeta M, Sisay Y (2016). Factors associated with prelacteal feeding in North Eastern Ethiopia: a community based cross-sectional study. Int Breastfeed J.

[9] Tariku A, Biks GA, Wassie MM, Gebeyehu A, Getie AA (2016). Factors associated with prelacteal feeding in the rural population of Northwest Ethiopia: a community cross-sectional study. Int Breastfeed J.

[10] Tadele N, Habta F, Akmel D, Deges E (2016). Knowledge, attitude and practice towards exclusive breastfeeding among lactating mothers in Mizan Aman town, Southwestern Ethiopia: descriptive cross-sectional study. Int Breastfeed J.

[11] Shifraw T, Worku A, Berhane Y (2015). Factors associated exclusive breastfeeding practices of urban women in Addis Ababa public health centers, Ethiopia: a cross sectional study. Int Breastfeed J.

[12] Dachew BA, Berhanu BB (2014). Breastfeeding practice and associated factors among female nurses and midwives at North Gondar zone, Northwest Ethiopia: a crosssectional institution based study. Int Breastfeed J.

[13] de Jager E, Broadbent J, Fuller-Tyszkiewicz M, Skouteris H (2014). The role of psychosocial factors in exclusive breastfeeding to six months postpartum. Midwifery..

[14] Meedya S, Fahy K, Kable A (2010). Factors that positively influence breastfeeding duration to 6 months: a literature review. Women Birth.

[15] Mutuli LA, Walingo MK, Othuon LA (2012). Assessing predictive power of psychosocial factors on breastfeeding behavior of mothers attending postnatal clinics in Western Kenya. Infant Child Adolesc Nutr.

[16] Minas AG, Ganga-Limando M (2016). Social-cognitive predictors of exclusive breastfeeding among primiparous mothers in Addis Ababa, Ethiopia. PLoS ONE.

[17] Wood NK, Woods NF, Blackburn ST, Sanders EA (2016). Interventions that enhance breastfeeding initiation, duration, and exclusivity: a systematic review. Am J Matern Child Nurs.

[18] Scott JA, Landers MC, Hughes RM, Binns CW (2001). Factors associated with breastfeeding at discharge and duration of breastfeeding. J Paediatr Child Health.

[19] Alive & Thrive (2010). Practices, IYCF practices, beliefs, and influences in Tigray Region, Ethiopia.

[20] USAID/ENGINE (2014). Fathers’ infant and young child feeding practices and their determinants in Amhara, Oromia, SNNP and Tigray Regions..

[21] Zhang J, Shi L, Chen D, Wang J, Wang Y (2009). Using the theory of planned behavior to examine effectiveness of an educational intervention on infant feeding in China. Prev Med.

[22] Gu Y, Zhu Y, Zhang Z, Wan H (2016). Effectiveness of a theory-based breastfeeding promotion intervention on exclusive breastfeeding in China: a randomised controlled trial. Midwifery..

[23] Zhu Y, Zhang Z, Ling Y, Wan H (2016). Impact of intervention on breastfeeding outcomes and determinants based on theory of planned behavior. Women Birth.

[24] Bich TH, Hoa DTP, Målqvist M (2014). Fathers as supporters for improved exclusive breastfeeding in Viet Nam. Matern Child Health J.

[25] Bich TH, Hoa DTP, Ha NT, Vui LT, Nghia DT, Målqvist M (2016). Father’s involvement and its effect on early breastfeeding practices in Viet Nam. Matern Child Nutr.

[26] Özlüses E, Çelebioglu A (2014). Educating fathers to improve breastfeeding rates and paternal-infant attachment. Indian Pediatr.

[27] Su M, Ouyang Y-Q (2016). Father’s role in breastfeeding promotion: lessons from a quasi-experimental trial in China. Breastfeed Med.

[28] Hector D, King L, Webb K, Heywood P (2005). Factors affecting breastfeeding practices: applying a conceptual framework. N S W Public Health Bull.

[29] Dennis CL (2002). Breastfeeding initiation and duration: a 1990-2000 literature review. J Obstet Gynecol Neonatal Nurs.

[30] Hennik M (2007). International focus group research.

[31] Greenbaum TL (1998). The handbook for focus group research.

[32] Gallegos D, Russell-Bennett R, Previte J (2011). An innovative approach to reducing risks associated with infant feeding : the use of technology. J Nonprofit Public Sect Mark.

[33] Hmone MP, Dibley MJ, Li M, Alam A (2016). A formative study to inform mHealth based randomized controlled trial intervention to promote exclusive breastfeeding practices in Myanmar: incorporating qualitative study findings. BMC Med Inform Decis Mak.

[34] Bai Y, Middlestadt SE, Peng CYJ, Fly AD (2007). Predictors of continuation of exclusive breastfeeding for the first six months of life. J Hum Lact.

[35] Belachew AB, Kahsay AB, Abebe YG (2016). Individual and community-level factors associated with introduction of prelacteal feeding in Ethiopia. Arch Public Health.

[36] Tadesse K, Zelenko O, Mulugeta A, Gallegos D (2018). Effectiveness of breastfeeding interventions delivered to fathers in low- and middle-income countries: a systematic review. Matern Child Nutr.

[37] Liamputtong P (2013). Qualitative research methods.

[38] Wilunda C, Quaglio G, Putoto G, Takahashi R, Calia F, Abebe D (2015). Determinants of utilisation of antenatal care and skilled birth attendant at delivery in South West Shoa Zone, Ethiopia: a cross sectional study. Reprod Health.

[39] Gebrehiwot H, Gebregziabher W, Gidey G (2013). Assessment of husbands’ participation on birth preparedness and complication readiness in Enderta Woreda, Tigray region, Ethiopia, 2012. J Women’s Health Care.

[40] Senarath U, Dibley MJ, Agho KE (2010). Factors associated with nonexclusive breastfeeding in 5 east and southeast Asian countries: a multilevel analysis. J Hum Lact.

[41] Negin J, Coffman J, Vizintin P, Raynes-Greenow C (2016). The influence of grandmothers on breastfeeding rates: a systematic review. BMC Pregnancy Childbirth.

[42] Chintalapudi N, Hamela G, Mofolo I, Maman S, Hosseinipour MC, Hoffman IF (2018). Infant and young child feeding decision making and practices: Malawian mothers’ and fathers’ roles in the context of HIV. J Hum Lact.

[43] Bilal S, Spigt M, Czabanowska K, Mulugeta A, Blanco R, Dinant G (2016). Fathers’ perception, practice, and challenges in young child care and feeding in Ethiopia. Food Nutr Bull.

[44] Rempel LA, Rempel JK (2011). The breastfeeding team: the role of involved fathers in the breastfeeding family. J Hum Lact.

[45] Tan KL (2011). Factors associated with exclusive breastfeeding among infants under six months of age in peninsular Malaysia. Int Breastfeed J.

[46] Dennis C-L (2003). The breastfeeding self-efficacy scale:psychometric assessment of the short form. J Obstet Gynecol Neonatal Nurs.

[47] Mogre V, Dery M, Gaa PK (2016). Knowledge, attitudes and determinants of exclusive breastfeeding practice among Ghanaian rural lactating mothers. Int Breastfeed J.

[48] Gatti L (2008). Maternal perceptions of insufficient milk supply in breastfeeding. J Nurs Scholarsh.

[49] Sacco LM, Caulfield LE, Gittelsohn J, Martinez H (2006). The conceptualization of perceived insufficient milk among Mexican mothers. J Hum Lact.

[50] McCarter-Spaulding DE, Kearney MH (2001). Parenting self-efficacy and perception of insufficient breast milk. J Obstet Gynecol Neonatal Nurs.

[51] Blyth R, Creedy D, Dennis C, Moyle W, Pratt J, De Vries S (2002). Effect of maternal confidence on breastfeeding duration: an application of breastfeeding self-efficacy theory. Birth..

[52] MacGowan RJ, MacGowan CA, Serdula MK, Lane JM, Joesoef RM, Cook FH (1991). Breast-feeding among women attending women, infants, and children clinics in Georgia, 1987. Pediatrics..

[53] Komodiki E, Kontogeorgou A, Papastavrou M, Volaki P, Genitsaridi S (2014). Breastfeeding in public: a global review of different attitudes towards it. J Pediatr Neonatal Care.

[54] Zhao Y, Ouyang YQ, Redding SR (2018). Chinese women's experiences, emotions and expectations of breast-feeding in public: a qualitative study. Public Health Nutr.

[55] Rempel LA, Rempel JK (2004). Partner influence on health behaviour decision making: increasing breast feeding duration. J Soc Pers Relat.

[56] Asemahagn MA (2016). Determinants of exclusive breastfeeding practices among mothers in azezo district, Northwest Ethiopia. Int Breastfeed J.

[57] Mekuria G, Edris M (2015). Exclusive breastfeeding and associated factors among mothers in Debre Markos, Northwest Ethiopia: a cross-sectional study. Int Breastfeed J.

[58] Liben ML, Gemechu YB, Adugnew M, Asrade A, Adamie B, Gebremedin E (2016). Factors associated with exclusive breastfeeding practices among mothers in dubti town, afar regional state, Northeast Ethiopia: a community based cross-sectional study. Int Breastfeed J.

[59] Seid AM, Yesuf ME, Koye DN (2013). Prevalence of exclusive breastfeeding practices and associated factors among mothers in Bahir Dar city, Northwest Ethiopia: a community based cross-sectional study. Int Breastfeed J.

